# Stem Cells Derived from Lipoma and Adipose Tissue—Similar Mesenchymal Phenotype but Different Differentiation Capacity Governed by Distinct Molecular Signature

**DOI:** 10.3390/cells7120260

**Published:** 2018-12-08

**Authors:** Sanja Stojanović, Stevo Najman, Aleksandra Korać

**Affiliations:** 1 Department of Biology and Human Genetics and Department for Cell and Tissue Engineering, Faculty of Medicine, University of Niš, 18000 Niš, Serbia; stevo.najman@medfak.ni.ac.rs; 2 Faculty of Biology, University of Belgrade, 11000 Belgrade, Serbia; aleksandra.korac@bio.bg.ac.rs

**Keywords:** lipomas, adipose tissue, stem cells, adipogenesis, osteogenesis

## Abstract

Lipomas are benign adipose tissue tumors of unknown etiology, which can vary in size, number, body localization and cell populations within the tissue. Lipoma-derived stem cells (LDSCs) are proposed as a potential tool in regenerative medicine and tissue engineering due to their similar characteristics with adipose-derived stem cells (ADSCs) reported so far. Our study is among the first giving detailed insights into the molecular signature and differences in the differentiation capacity of LDSCs in vitro compared to ADSCs. Mesenchymal stem cell phenotype was analyzed by gene expression and flow cytometric analysis of stem cell markers. Adipogenesis and osteogenesis were analyzed by microscopic analysis, cytochemical and immunocytochemical staining, gene and protein expression analyses. We showed that both LDSCs and ADSCs were mesenchymal stem cells with similar phenotype and stemness state but different molecular basis for potential differentiation. Adipogenesis-related genes expression pattern and presence of more mature adipocytes in ADSCs than in LDSCs after 21 days of adipogenic differentiation, indicated that differentiation capacity of LDSCs was significantly lower compared to ADSCs. Analysis of osteogenesis-related markers after 16 days of osteogenic differentiation revealed that both types of cells had characteristic osteoblast-like phenotype, but were at different stages of osteogenesis. Differences observed between LDSCs and ADSCs are probably due to the distinct molecular signature and their commitment in the tissue that governs their different capacity and fate during adipogenic and osteogenic induction in vitro despite their similar mesenchymal phenotype.

## 1. Introduction

Adipose-derived stem cells (ADSCs) are adult mesenchymal stem cells (MSCs) originated from adipose tissue that show a great morphological and functional similarity with MSCs from bone marrow but with additional advantages [1]. ADSCs can be isolated from abundant and easily accessible adipose tissue in large quantities with a minimal invasive procedure either from liposuction aspirates or adipose tissue biopsies [2] and have properties that make them good candidates for regenerative medicine applications [3]. The use of ADSCs in tissue engineering and regenerative medicine is very promising due to their self-renewal potential, proliferation capacity and great potential to differentiate into numerous cell types particularly adipocytes, osteoblasts, chondrocytes and endothelial cells [4,5,6,7]. It is reported that ADSCs can be successfully differentiated into the cells of all three germ lines which means that they can be considered as pluripotent and makes them a good candidate for wide range applications in the field of biomedical sciences [4].

Lipomas are benign tumors of adipose tissue and represent one of the most common soft tissue neoplasms of mesenchymal origin [8,9]. Lipomas can be solitary, multiple generalized lipomatosis and multiple symmetric, usually slowly growing, diffused or encapsulated, can vary in size and shape and show great heterogeneity in cell populations presented within the tissue [10,11]. The occurrence of lipomas in all body parts has been reported, mostly in subcutaneous depots but also in other tissues, organs and body cavities. Both male and female can be affected and all ages although they usually appear in the middle age [9,12]. Several studies reported that lipoma tissue is a good source of stem cells that might be used for regenerative medicine purposes, naming lipoma “useless tissue useful in the application of regenerative medicine and tissue engineering” [13]. Isolation and characterization of MSCs from lipoma, so called lipoma-derived stem cells (LDSCs) was reported and it has been shown that those cells are very similar to ones isolated from normal adipose tissue [14]. It has been shown that LDSCs can proliferate in a similar manner as ADSCs, express characteristic mesenchymal stem cell markers and can differentiate into adipocytes, osteoblasts and chondrocytes like ADSCs [13,15,16]. There are, however, only few publications and studies that dealt with some of the LDSCs properties. In almost all publications authors showed that LDSCs have the same potential to differentiate into osteoblasts and adipocytes as ADSCs with very few reports on different proliferation potential and characteristics of LDSCs compared to ADSCs [17]. Also, there are reports about formation of bone and cartilaginous structures within lipoma tissue and its ossification in various parts of the body [18], with considerations that stem cells from lipoma tissue may be responsible for those processes [19]. 

Bearing in mind that lipomas are adipose tissue tumors with insufficiently clarified etiology and pathogenesis, and that transformations of lipoma tissue into hard tissue structures can occur, which implies the role of LDSCs, as well as potential use of LDSCs in regenerative purposes reported so far and lack of detailed comparison with ADSCs; the request and the need for further investigation on molecular and cellular features of LDSCs arises, as potential cause of lipoma formation and their possible application in regenerative medicine, as well as detailed comparison with ADSCs from normal adipose tissue. To the best of our knowledge, our study is among the first giving detailed insights into the molecular signature of LDSCs, with the first data on adipogenic- and osteogenic-related markers’ expression and comparison with ADSCs, as well as showing that LDSCs and ADSCs have different characteristics particularly in the differentiation capacity in vitro. Here we presented the cellular and molecular features and comparison of those two types of MSCs with special emphasis on adipogenic and osteogenic differentiation capacity.

## 2. Materials and Methods

### 2.1. Patients

Lipoma tissue samples were obtained at surgical clinics of the Clinical Center Niš, Serbia after surgical removal of solitary subcutaneous lipomas that were clinically and pathologically diagnosed as lipoma and distinguished from other adipose tissue neoplasms. Subcutaneous adipose tissue samples were obtained from non-cancer patients during other surgeries. All patients gave their informed written consent and the study was approved by the Local Ethical Committee of the Faculty of Medicine, University of Niš, Serbia (approvals no. 01-6481-15 and 12-6316-2/4). Tissue sample biopsies from 14 patients were analyzed, among them 8 lipomas and 6 normal adipose tissue samples. Average age of patients with lipoma was 48.3 ± 8.3 while average age of non-lipoma patients was 49.5 ± 11.1. In the group of patients with lipoma, 5 were female and 3 were male, while in the non-lipoma group of patients 4 were female and 2 were male. Lipomas and adipose tissue samples were taken from several subcutaneous body depots: upper arm, back, neck, abdomen, hip and thigh. Body mass index (BMI) for all patients was less than 30, indicated non-obese patients.

### 2.2. Isolation and Cultivation of Mesenchymal Stem Cells

Both lipoma-derived stem cells (LDSCs) and adipose-derived stem cells (ADSCs) were isolated by enzymatic digestion of tissue samples, respectively. Tissue samples were washed, cut into small pieces and placed in 0.1% collagenase type I solution (StemCell Technologies, Vancouver, CO, Canada), in a water bath at 37 °C for 45 min. Tissue homogenates were then vortex and filtered and cell culture media was added to stop collagenase. Stromal vascular fraction (SVF) of cells was obtained by centrifugation for 15 min at 1500 rpm, collected from the bottom of the tube and seeded in 25 cm^2^ cell culture flask (Greiner Bio One, Kremsmünster, Austria) in standard cell culture medium (DM) that contained Dulbecco’s Modified Eagle’s medium (DMEM), 10% fetal bovine serum (FBS), 2 mM stable glutamine and 1% antibiotic-antimycotic solution (all purchased from Capricorn Scientific, Ebsdorfergrund, Germany). Cells were washed and media was changed 16–18 h after isolation to remove non-attached cells. After reaching the 70–80% confluency, first cell passage was performed (P1), which enabled purification of mesenchymal stem cells. Cells were cultured in standard cell culture conditions which mean temperature of 37 °C and humidified atmosphere with the presence of 5% CO_2_. Medium was changed every three days.

### 2.3. Differentiation of Cells 

For differentiation assays, cells at passage 2 (P2) were used. Cells were passaged by using 0.25 U/mL dispase in DMEM/F-12 (StemCell Technologies, Vancouver, CO, Canada) and 0.05% trypsin-EDTA solution (Capricorn Scientific, Ebsdorfergrund, Germany), centrifuged and cell number was determined by Trypan blue dye exclusion assay on Countess™ automated cell counter (Thermo Scientific, Waltham, MA, USA). For adipogenic differentiation assay, 5000 cells per cm^2^ were seeded onto sterile glass coverslips in 24-well culture plates (Greiner Bio One, Kremsmünster, Austria) and left to attach overnight. Medium in which cells were seeded was then replaced with adipocyte differentiation media (AM) which was purchased from Gibco^®^ (Carlsbad, CA, USA), (StemPro™ Adipogenesis Differentiation Kit) or standard cell culture media (DM). Cells were cultured in AM and DM media up to 21 days. Media were changed every 3 to 4 days. For osteogenic differentiation assay, 3000 cells per cm^2^ were seeded onto sterile glass coverslips in 24-well culture plates and left to attach overnight. Medium in which cells were seeded was then replaced with osteogenic differentiation media (OS) or standard cell culture media (DM). Osteogenic differentiation media was prepared by adding 0.1 µM of dexamethasone (D4902, Sigma, St. Louis, MO, USA), 50 µM l-ascorbic acid 2-phosphate (49752, Sigma) and 2 mM β-Glycerophosphate (AppliChem, Darmstadt, Germany) to the DM medium. Cells were cultured in OS and DM media up to 16 days. Media were changed every 3 to 4 days in both assays and cells were cultured in standard cell culture conditions.

### 2.4. Light Microscopy

Cells after isolation, during differentiation studies and after staining were monitored on inverted light microscope (Observer Z1, Carl Zeiss, Oberkochen, Germany), under phase contrast and bright field. The images were acquired using the camera AxioCam HR (Carl Zeiss, Germany) and the software ZEN 2 blue edition (Carl Zeiss, Germany).

### 2.5. Flow Cytometry

Expression of mesenchymal stem cell surface marker CD105 was analyzed on both LDSCs and ADSCs, respectively, at P2 (before differentiation assays). After trypsinization, centrifugation and washing, cells were stained for 15 min with PE conjugated monoclonal mouse anti-CD105 human antibody (Clone 43A4E1, Miltenyi Biotec, Bergisch Gladbach, Germany) at 4 °C. After washing steps, cells were re-suspended in a buffer and analyzed on BD LSRFortessa™ cell analyzer with BD FACSDiva™ software v8 (BD Biosciences, Heidelberg, Germany). A total of minimum 100,000 cells were usually acquired for each sample.

### 2.6. RNA Isolation and Reverse Transcription

LDSCs and ADSCs at passage 2 (day 0 in differentiation assay), at day 21 in adipogenic differentiation assay and days 8 and 16 in osteogenic differentiation assay were placed in RNAlater^®^ (Ambion, Life Technologies, Carlsbad, CA, USA) and stored at −80°C until RNA purification step. The total RNA was isolated from the cells using RNeasy Mini Kit (Qiagen, Venlo, The Netherlands) according to the manufacturer’s instructions. DNase I RNase-free set (Qiagen) was used for on-column digestion of residual genomic DNA, according to the manufacturer’s instructions. The RNA concentration was determined immediately after isolation using Qubit™ RNA HS Assay Kit (Thermo Scientific, Waltham, MA, USA) on Qubit^®^ 2.0 fluorimeter (Invitrogen, Thermo Scientific, Waltham, MA, USA), according to the manufacturer’s instructions. Total RNA was reversely transcribed into single-stranded cDNA using High-capacity cDNA Reverse Transcription Kit (Applied Biosystems^®^, Foster City, CA, USA), according to the manufacturer’s instructions, with 100 ng per reaction per sample. Reverse transcription was performed in the PCR thermal cycler SureCycler8800 (Agilent Technologies, Santa Clara, CA, USA). The protocol conditions were: 10 min at 25 °C, 120 min at 37 °C, 5 min at 85°C and cooling at 4 °C. The synthesized cDNA was stored at −80 °C and was later used as a template for qPCR to determine the relative gene expression.

### 2.7. Real Time PCR

Quantitative real time PCR reactions were performed by real time thermal cycler Stratagene Mx3005P (Agilent Technologies, Santa Clara, CA, USA). The qPCR reactions were prepared by using SYBR Fast Universal 2x qPCR Master Mix (Kapa Biosystems, Wilmington, MA, USA), according to the manufacturer’s instruction. ROX was used as a reference dye. Pre-designed primer sets (QuantiTect primer assay kits) were purchased from Qiagen, The Netherlands. Primer kits, consisted of both forward and reverse primers, were used for the following genes: *GAPDH* (QT00079247), *CD44* (QT00073549), *POU5F1* (QT00210840), *BGLAP* (QT00232771), *RUNX2* (QT00998102), *DLK1* (QT00093128), *PPARG* (QT00029841), *LEP* (QT00030261), and *ADIPOQ* (QT00014091). The protocol conditions were: (1) enzyme activation: 3 min at 95 °C (1 cycle); (2) denaturation: 3 s at 95 °C and annealing/extension (with data acquisition): 30 s at 60 °C (40 cycles). The specific binding of primers was confirmed by melting curve analysis and specific length product visualization on electrophoresis gel. The expression level of each target gene was normalized to the glyceraldehyde-3-phosphate dehydrogenase housekeeping gene expression (*GAPDH*) in the same sample. The relative gene expression data analysis was performed by the relative quantification method 2^–∆∆Ct^ as described by Livak and Schmittgen [20]. Human XpressRef Universal Total RNA (338112, Qiagen) was used as calibrator for all qPCR reactions.

### 2.8. Oil Red O Staining

To assess the mature adipocytes’ formation after 21 days of adipogenic differentiation, the presence of lipid droplets was analyzed by Oil Red O staining. LDSCs and ADSCs cells were fixed in 10% neutral buffered formalin (NBF), washed, incubated for 3 min in 60% isopropanol and then Oil Red O solution was applied for 15 min. After washing, cells were counterstained with Mayer’s hematoxylin (Bio-Optica, Milan, Italy) for 1 min and imaged. Quantification of lipid droplets was performed by dissolving the Oil red O dye in 100% isopropanol and measuring absorbance at 450 nm on multichannel spectrophotometer (Multiskan Ascent plate reader, ThermoLab Systems, Helsinki, Finland).

### 2.9. Cytochemical Staining for Calcium Deposits

For the detection of calcium deposits in cells underwent osteogenic differentiation, Alizarin red S (ARS) and Von Kossa staining was performed. After 16 days of osteogenic differentiation, LDSCs and ADSCs were fixed in 10% NBF. Alizarin red S (A5533, Sigma-Aldrich, St. Louis, MO, USA) staining solution at concentration 2% was applied to the cells and incubated for 45 min at room temperature (RT) in the dark. Cells were then washed and analyzed under the microscope. Quantification of inorganic deposits was performed by dissolving ARS in 10% acetic acid solution and absorbance measurement at 405 nm on plate reader. For Von Kossa staining, 2% silver nitrate solution was applied to the fixed cells and exposed to the UV light for 30 min at RT. After washing cells were treated with 5% sodium thiosulfate solution for 5 min at RT, counterstained with Nuclear Fast Red Solution (N069.1, Carl Roth, Karlsruhe, Germany) for 5 min and mounted with DPX mounting medium (06522, Sigma).

### 2.10. Immunocytochemistry

Immunoexpression of adiponectin in LDSCs and ADSCs differentiated for 21 day toward adipocytes was analyzed by immunocytochemical staining. Cells were fixed in 10% NBF for 15 min, washed and endogenous peroxide and protein block were applied. Anti-adiponectin antibody ([19F1], ab22554, Abcam, Cambridge, UK) was applied on cells at dilution 1:400 and incubated over night at +4 °C. For visualization, rabbit specific HRP/DAB (ABC) detection IHC kit (ab64261, Abcam, Cambridge, UK) was used according to the manufacturer’s protocol. Cells were counterstained with Mayer’s hematoxylin and mounted with VectaMount Permanent Mounting Medium (Vector Laboratories, Peterborough, UK).

### 2.11. ELISA Assays

Measurement of secretion products in cell culture media of LDSCs and ADSCs differentiated into adipocytes and osteoblasts was performed by ELISA assays. Level of osteoprotegerin, as osteoblast marker, was analyzed at day 16 of osteogenic differentiation while level of leptin, as mature adipocyte marker, was analyzed at day 21 of adipogenic differentiation in cell culture supernatant. Both leptin and osteoprotegerin measurements were performed using 72 h conditioned media. Human Osteoprotegerin ELISA Kit (ab100617) was purchased from Abcam, UK while Human Leptin Quantikine ELISA Kit (DLP00) was purchased from RnD systems (Minneapolis, MN, USA). Both ELISAs were performed according to the manufacturer’s instructions, respectively. Values are expressed as pg of leptin or osteoprotegerin per ml.

### 2.12. Statistical Analysis

Results of real time PCR analyses and ELISA assays are presented as scatterplots with median using the templates published by Weissgerber et al. [21]. All the results are statistically processed and for all samples median as well as mean values were calculated. Mean values are presented with standard deviation (SD). Data were analyzed by one-way ANOVA or Mann–Whitney U-test to compare and determine statistically significant differences between the samples. The value of *p* < 0.05 was considered as significant. 

## 3. Results

### 3.1. Analysis of Mesenchymal Stem Cell Phenotype

**Figure 1:**
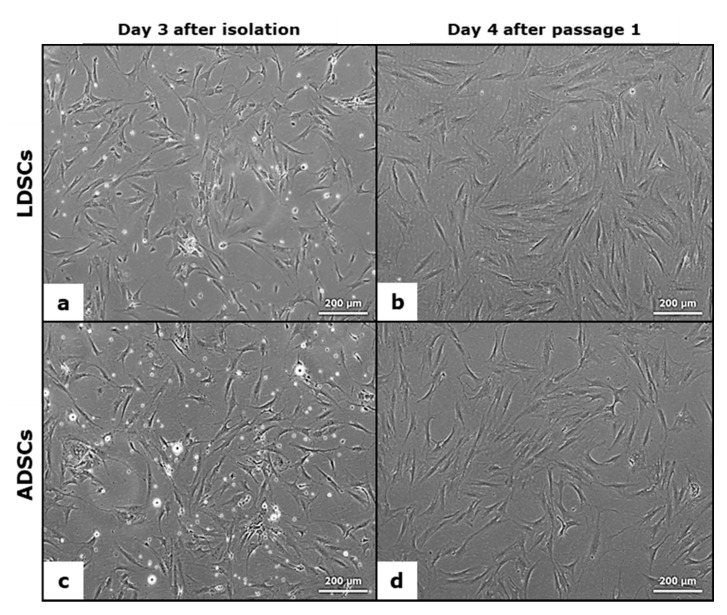
Morphology of lipoma-derived stem cells (LDSCs) (**a**,**b**) and adipose-derived stem cells (ADSCs) (**c**,**d**) cultures; Images were acquired at day 3 after isolation (**a**,**c**) and at day 4 after passage 1 (**b**,**d**); phase contrast with objective magnification 10×; cells are spindle-like in shape which is typical for mesenchymal stem cells.

In Figure 1 the morphology of LDSCs (a,b) and ADSCs (c,d) is presented. There were no differences in morphology between LDSCs and ADSCs either at day 3 after isolation (Figure 1a,c) or at passage 1 day 4 (Figure 1b,d).

**Figure 2:**
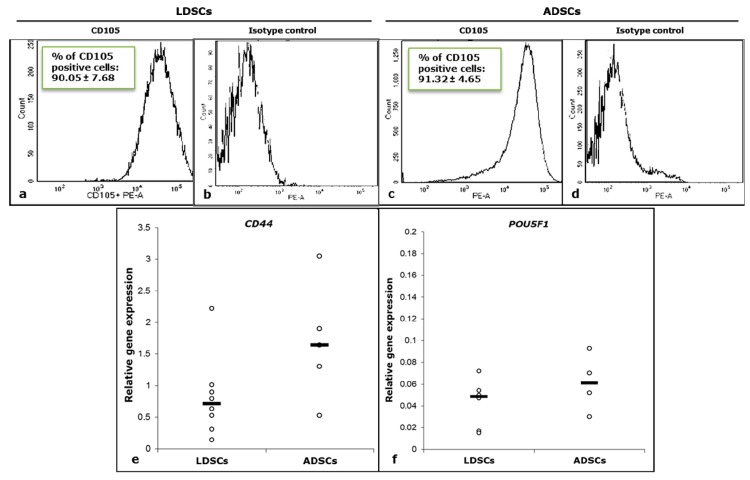
Flow cytometric analysis of CD105 cell surface marker expression in LDSCs (**a**) and ADSCs (**c**) at passage 2 (representative histograms per each group of samples with % of CD105 positive cells presented as mean ± SD, n (LDSCs) = 6 and n (ADSCs) = 4); corresponding isotype controls (**b**,**d**); Relative expression of *CD44* (**e**) and *POU5F1* (**f**) genes in LDSCs and ADSCs at passage 2 (day 0 in differentiation assays), normalized to *GAPDH*, presented as scatterplots with median; sample size for *CD44*: n (LDSCs) = 8 and n (ADSCs) = 5, for *POU5F1*: n (LDSCs) = 6 and n (ADSCs) = 4.

Flow cytometric analysis (Figure 2a–d) showed high expression of surface stem cell marker CD105 in both LDSCs (Figure 2a) and ADSCs (Figure 2c) at passage 2, just before differentiation assays. Non-specific antibody binding was excluded by using isotype control (Figure 2b,d). 

Real time PCR analysis of *CD44* and *POU5F1* stem cell markers’ expression (Figure 2e,f) confirmed that both LDSCs and ADSCs express these genes at passage 2. Slightly higher expression of *CD44* in ADSCs compared to LDSCs (Figure 2e) was not statistically significant (*p* = 0.1).

**Figure 3:**
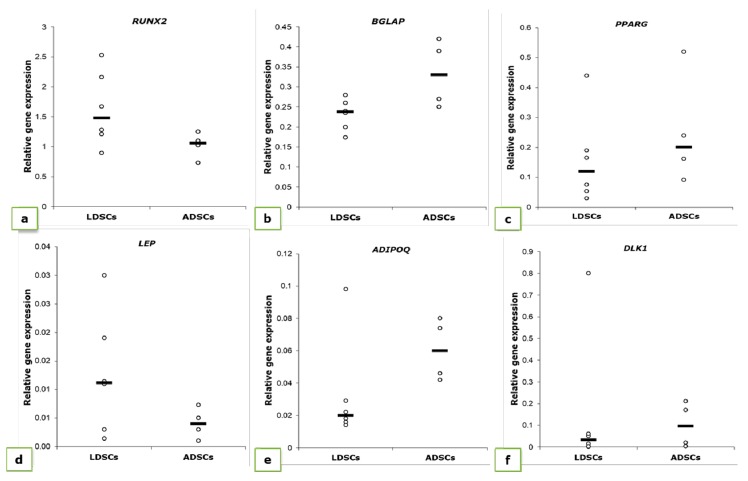
Relative expression of *RUNX2* (**a**), *BGLAP* (**b**), *PPARG* (**c**), *LEP* (**d**), *ADIPOQ* (**e**) and *DLK1* (**f**) genes in LDSCs and ADSCs at passage 2 (day 0 in differentiation assays), normalized to *GAPDH*; significant difference between cells was noticed for *RUNX2* and *BGLAP* expression (*p* < 0.05); scatterplots with median; n (LDSCs) = 6 and n (ADSCs) = 4 for all genes.

We also analyzed expression levels of genes characteristically expressed during osteogenesis (*RUNX2* and *BGLAP*) and adipogenesis (*ADIPOQ, LEP, PPARG* and *DLK1*) in cells at passage 2, day 0 in differentiation studies (Figure 3). Differences in expression levels of all examined genes were noticed between LDSCs and ADSCs, but statistically significant only for *RUNX2* and *BGLAP*. *RUNX2* expression was higher in LDSCs compared to ADSCs while *BGLAP* expression was higher in ADSCs compared to LDSCs (*p* < 0.05).

### 3.2. Adipogenic Differentiation

**Figure 4:**
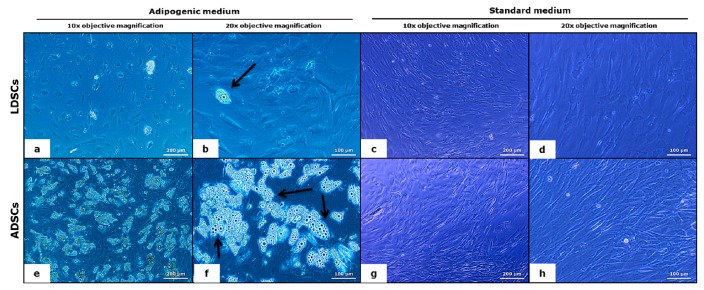
Light microscopic analysis of adipocytes’ formation after 21 day of adipogenic differentiation; LDSCs in adipogenic medium (AM) (**a**,**b**), LDSCs in standard medium (DM) (**c**,**d**), ADSCs in AM medium (**e**,**f**) and ADSCs in DM medium (**g**,**h**); phase contrast with objective magnification 10× (**a**,**c**,**e**,**g**) and 20× (**b**,**d**,**f**,**h**); arrows indicate mature adipocytes.

Adipogenic differentiation of both LDSCs and ADSCs was analyzed after 21 days of cultivation in adipogenic medium (AM). As control, cells were cultivated in standard medium (DM) under the same conditions. Characteristic adipocyte-like phenotype and the presence of lipid droplets were noticed in both LDSCs (Figure 4a,b) and ADSCs (Figure 4e,f) after 21 days of differentiation. However, lipid droplets were noticeably significantly more present in the ADSCs (Figure 4e,f) compared to LDSCs (Figure 4a,b) which indicates higher adipogenic potential of ADSCs compared to LDSCs. Further, changes in the morphology, from mesenchymal to epithelial-like, characteristic for adipocytes, were noticed in both ADSCs and LDSCs culture. Cells cultivated in medium DM retained their mesenchymal-like and fibroblastic-like shape, very similar in both LDSCs (Figure 4c,d) and ADSCs (Figure 4g,h).

**Figure 5:**
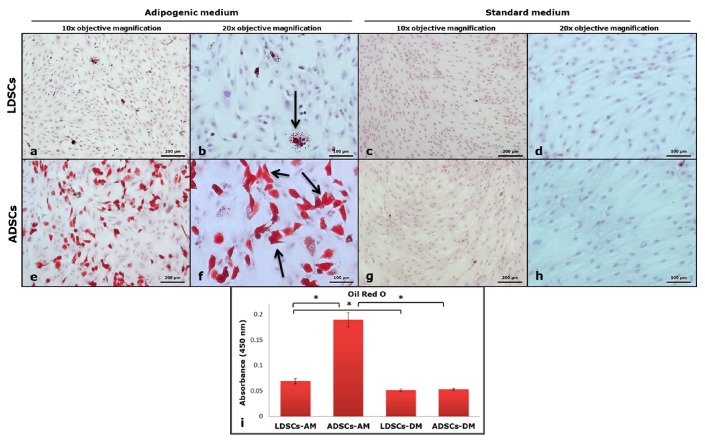
Oil red O staining of cells after 21 day of adipogenic differentiation; LDSCs in adipogenic medium (AM) (**a**,**b**), LDSCs in standard medium (DM) (**c**,**d**), ADSCs in AM medium (**e**,**f**) and ADSCs in DM medium (**g**,**h**); bright field with objective magnification 10× (**a**,**c**,**e**,**g**) and 20× (**b**,**d**,**f**,**h**); arrows indicate accumulation of lipids in cells; quantitative measurement of Oil red O dye (**i**), presented as mean ± SD, n = 4 for all groups; (*) *p* < 0.05.

**Figure 6:**
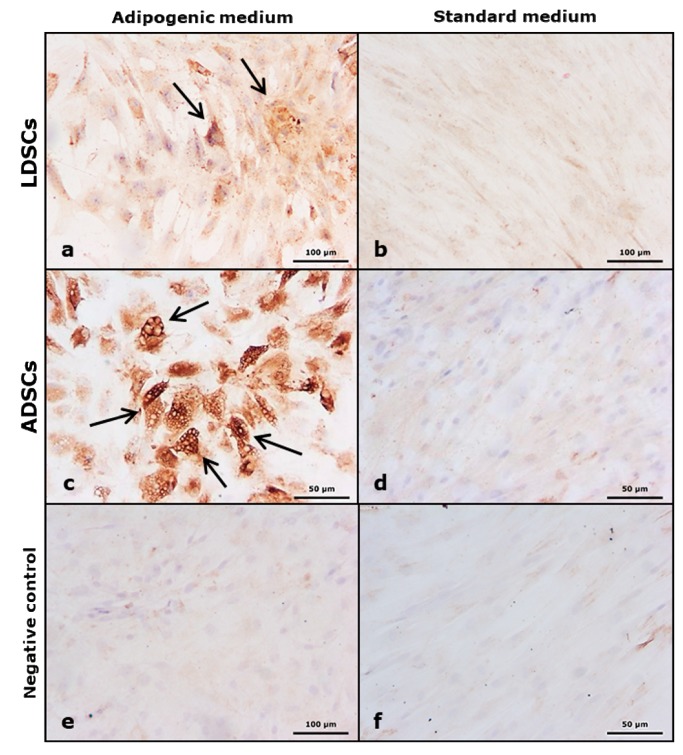
Immunoexpression of adiponectin in cells after 21 day of adipogenic differentiation; LDSCs in adipogenic medium (AM) (**a**), LDSCs in standard medium (DM) (**b**), ADSCs in AM medium (**c**) and ADSCs in DM medium (**d**); cells stained without primary antibody (non-specific background staining control – negative control) in AM medium (**e**) and in DM medium (**f**); bright field with objective magnification 20×; arrows indicate positive adiponectin immunostaining (brown).

After 21 days of adipogenic differentiation, presence of lipids was confirmed by Oil red O staining (Figure 5). Significantly more lipids were accumulated in ADSCs culture (Figure 5e,f) than LDSCs culture (Figure 5a,b), as confirmed by quantification of Oil red O dye (Figure 5i), indicating higher adipogenic differentiation potential of ADSCs. This was confirmed by adiponectin immunostaining after 21 days of adipogenic differentiation of ADSCs and LDSCs (Figure 6). Although both ADSCs and LDSCs were positive for adiponectin, much stronger staining was observed in ADSCs (Figure 6a,c). Neither LDSCs nor ADSCs cultivated in DM medium were positive for adiponectin excluding possibility of spontaneous adipogenesis (Figure 6b,d). Non-specific staining was not present, indicated by control staining omitting primary antibody (Figure 6e,f).

**Figure 7:**
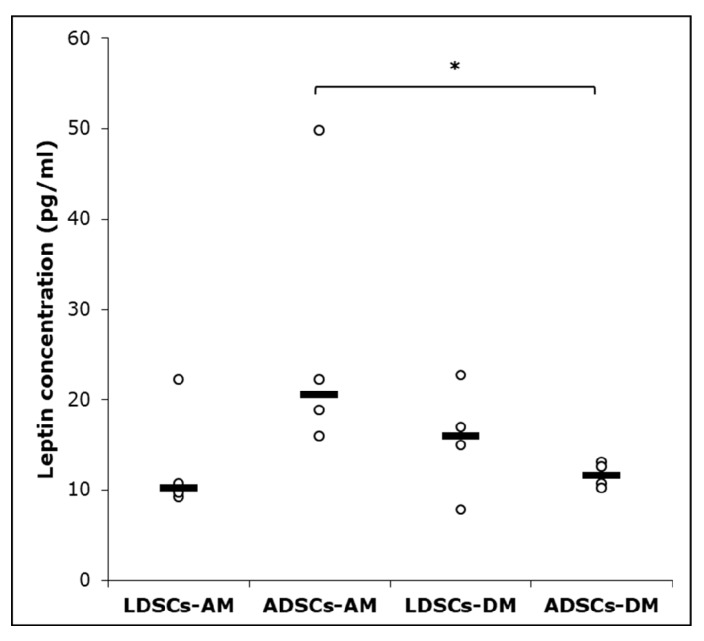
Leptin concentration in cell culture supernatant of LDSCs and ADSCs measured by ELISA at day 21 of adipogenic differentiation; scatterplots with median; n = 4 for all groups; (*) *p* < 0.05.

After 21 days of adipogenic differentiation, leptin concentration was measured in cell culture supernatant, by ELISA assay (Figure 7). Significantly higher concentration of leptin was observed in ADSCs in AM than in DM medium (*p* < 0.05), but no significant difference in leptin secretion was observed in LDSCs during differentiation. Leptin concentration was higher, but not statistically significant, in ADSCs than in LDSCs cultured in AM medium.

**Figure 8:**
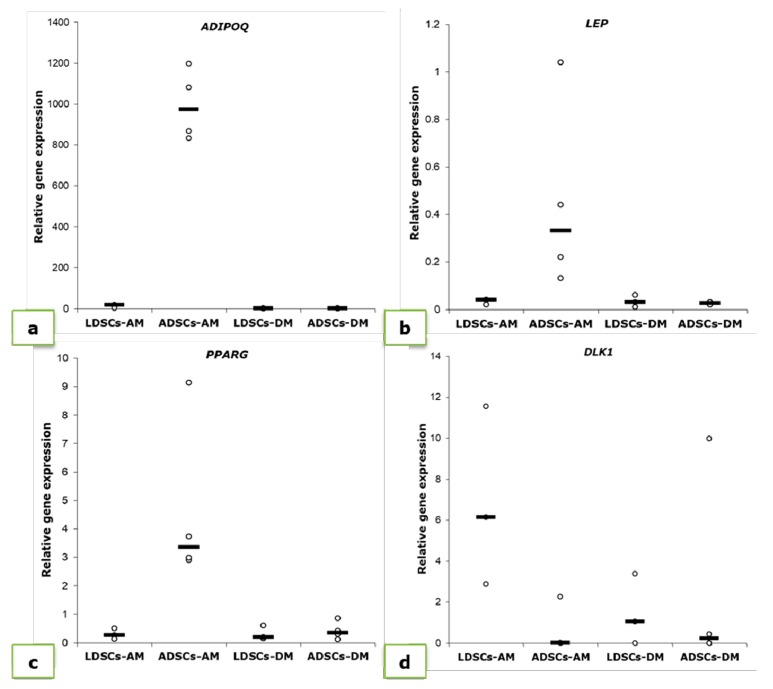
Relative expression levels (2^-ΔΔCt^) of genes for adiponectin (*ADIPOQ*) (**a**), leptin (*LEP*) (**b**), PPAR-gamma (*PPARG*) (**c**) and Pref-1 (*DLK1*) (**d**), measured in LDSCs and ADSCs at day 21 of adipogenic differentiation and normalized to *GAPDH*; scatterplots with median; n = 4 for all groups and genes.

We performed quantitative real time PCR to determine the expression levels of adipogenesis-related genes: adiponectin (*ADIPOQ*), leptin (*LEP*), PPAR-gamma (*PPARG*) and Pref-1 (*DLK1*), after 21 days of adipogenic differentiation (Figure 8, Table 1). As expected, expression of mature adipocytes’ markers *ADIPOQ*, *LEP* and *PPARG* increased in differentiated ADSCs (Figure 8a–c; Table 1) statistically significant (*p* < 0.05) while expression of *DLK1*, characteristic marker of pre-adipocytes, decreased (Figure 8d; Table 1). Expression levels of *ADIPOQ* and *LEP* slightly increased in LDSCs during differentiation (*p* < 0.05 only for *ADIPOQ*), but at much lower extent than in ADSCs. No significant changes in *PPARG* expression were observed in LDSCs (Figure 8c; Table 1) while *DLK1* expression significantly increased in LDSCs during differentiation (Figure 8d; Table 1). Cells cultivated in DM media did not show significant increase in adipogenic genes expression (Figure 8, Table 1).

### 3.3. Osteogenic Differentiation

**Figure 9:**
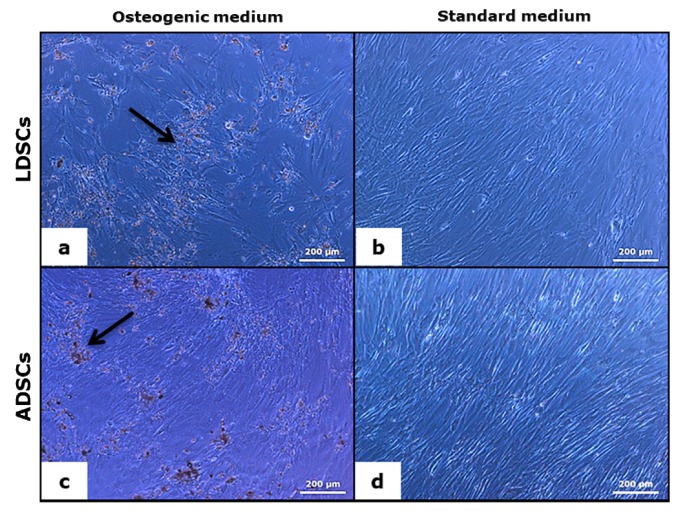
Light microscopic analysis of osteoblasts’ formation after 16 days of osteogenic differentiation; LDSCs in osteogenic medium (OS) (**a**) LDSCs in standard medium (DM) (**b**) ADSCs in OS medium (**c**) and ADSCs in DM medium (**d**); phase contrast with objective magnification 10×; arrows indicate inorganic material deposition.

Osteogenic differentiation of both LDSCs and ADSCs was analyzed after 8 and 16 days of cultivation of cells in OS medium. As control, cells were cultivated in medium DM under the same conditions. Characteristic osteoblast-like phenotype and accumulation of inorganic material were noticed at day 16 in both LDSCs and ADSCs cultured in OS medium, as observed by light microscopy (Figure 9a,c). The cells cultivated in medium DM retained their mesenchymal-like and fibroblastic-like shape, very similar in both LDSCs (Figure 9b) and ADSCs (Figure 9d).

**Figure 10:**
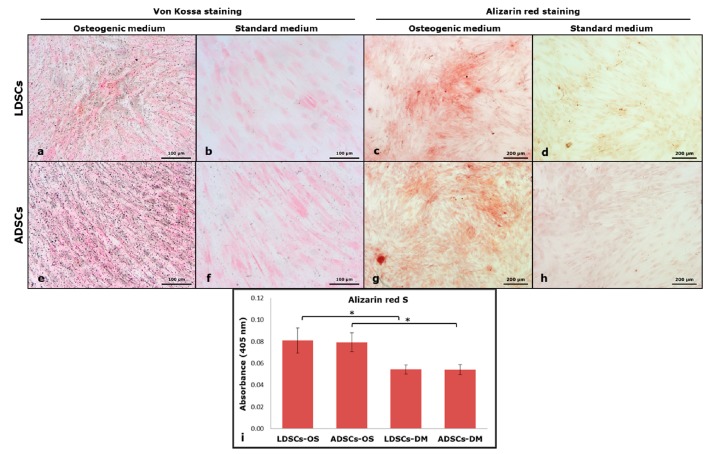
Von Kossa staining of cells at day 16 of osteogenic differentiation; LDSCs in osteogenic medium (OS) (**a**) LDSCs in standard medium (DM) (**b**) ADSCs in OS medium (**e**) and ADSCs in DM medium (**f**); dark grey/black deposits’ color in (**a**) and (**e**) indicates positive staining; Alizarin red S staining of cells at day 16 of osteogenic differentiation; LDSCs in OS medium (**c**) LDSCs in DM medium (**d**) ADSCs in OS medium (**g**) and ADSCs in DM medium (**h**); red deposits’ color in (**c**) and (**g**) indicates positive Alizarin red S (ARS)staining; All images were acquired at bright field with objective magnification 10×; quantitative measurement of ARS dye (**i**), presented as mean ± SD, n (LDSCs) = 5 and n (ADSCs) = 4 for all groups; (*) *p* < 0.05.

Inorganic deposits were stained by Von Kossa and Alizarin red S staining methods (Figure 10). Dark grey/black deposits in LDSCs (Figure 10a) and ADSCs (Figure 10e), indicating positive Von Kossa staining, and red colored deposits, indicating positive ARS staining, in LDSCs (Figure 10c) and ADSCs (Figure 10g) were noticed after 16 days in OS media. No colored deposits were observed in DM media neither in LDSCs nor ADSCs, after Von Kossa (Figure 10b,f) and ARS staining (Figure 10d,h).

**Figure 11:**
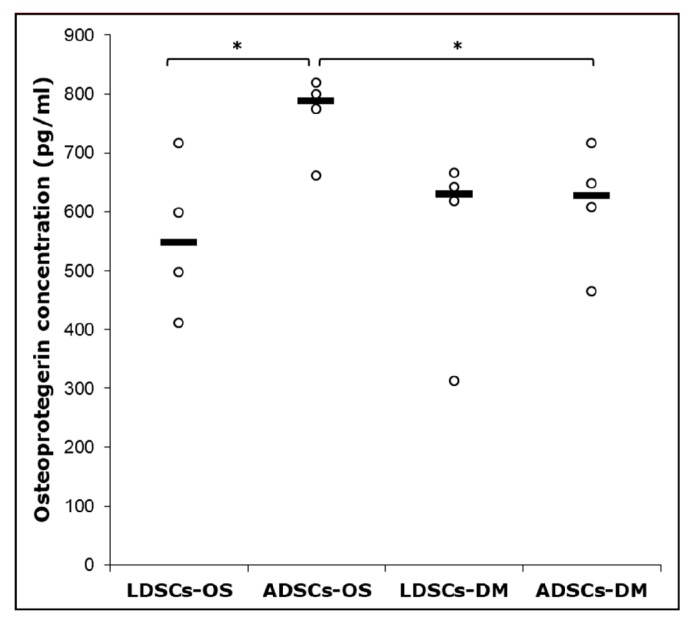
Osteoprotegerin concentration in cell culture supernatant of LDSCs and ADSCs, measured by ELISA at day 16 of osteogenic differentiation; scatterplots with median; n = 4 for all groups; (*) *p* < 0.05.

After 16 days of osteogenic differentiation, osteoprotegerin (OPG) concentration was measured in cell culture supernatant by ELISA (Figure 11). Significantly higher concentration of OPG (*p* < 0.05) was observed in ADSCs cultured in OS medium than in standard DM medium, and ADSCs than in LDSCs cultured in OS medium. No statistical significance was found between LDSCs cultured in OS compared to DM medium.

**Figure 12:**
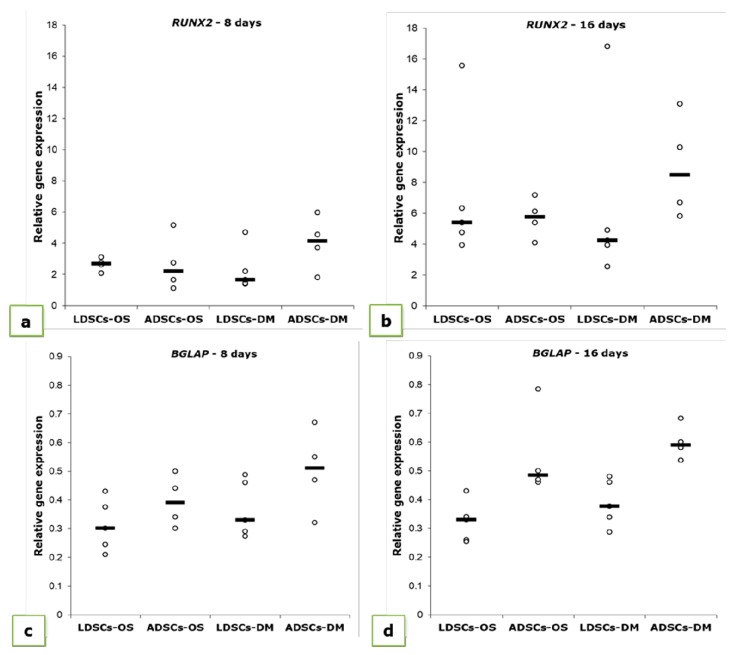
Relative expression levels (2^-ΔΔCt^) of *RUNX2* (**a**,**b**) and *BGLAP* (**c**,**d**) measured in LDSCs and ADSCs at day 8 (**a**,**c**) and day 16 (**b**,**d**) of osteogenic differentiation and normalized to *GAPDH*; scatterplots with median; n (LDSCs) = 5 and n (ADSCs) = 4 for all groups.

Relative expression levels of osteogenesis-related genes: runt related transcription factor 2 (*RUNX2*) and osteocalcin (*BGLAP*) were measured at two time points during osteogenic differentiation, 8 and 16 days (Figure 12, Table 2). After 8 days, the increase in *RUNX2* expression was observed in both LDSCs and ADSCs, but more pronounced in ADSCs than in LDSCs (Table 2). After 16 days of differentiation, *RUNX2* expression pattern has changed so that expression was higher in LDSCs cultured in OS than in DM medium, while lower in ADSCs cultured in OS than in DM medium (Figure 12b, Table 2). *BGLAP* expression slightly increased during differentiation and it was significantly higher in ADSCs than in LDSCs at both time points.

## 4. Discussion

Our study is among the first giving detailed analysis of stem cells isolated from lipoma and comparison with stem cells isolated from normal adipose tissue. The distinct signature of these cells governs the capacity for adipogenic and osteogenic differentiation which we analyzed on cellular and molecular levels.

Lipomas, used in our study, were located subcutaneously at different body locations. To compare cells isolated from lipoma with the cells isolated from normal adipose tissue, we used samples of subcutaneous adipose tissue from different body depots as well. It was reported that there is no statistically significant evidence of depot-related effects on proliferation and differentiation potential of ADSCs, while difference was noticed in apoptosis susceptibility and lipolysis [22]. Other study has shown that subcutaneous ADSCs, compared with ADSCs from other depots, have greater differentiation potential and recommend them as optimal choice for angiogenic and osteogenic regeneration [23]. Regarding the impact of donor age on the properties of ADSCs, results found in published data are diverse. Some studies showed that there are age-related differences in proliferation of ADSCs and lipid accumulation during adipogenic differentiation, with decrease in proliferation rate and increased lipid accumulation during aging [22]. Others reported that growth kinetics, osteogenic and chondrogenic differentiation potentials of ADSCs were adversely affected by increased donor age while adipogenic differentiation potential was maintained during aging [24]. On the other side, it is reported that the use of adipose stem cells for bone tissue engineering is not limited by the donor’s age as shown by osteogenic differentiation of ADSCs isolated from differently aged patients, on the scaffold-free 3D osteogenic graft intended for the treatment of bone defects [25]. In our study, both groups of patients were of similar age (average age of patients with lipoma was 48.3 ± 8.3 while average age of non-lipoma patients was 49.5 ± 11.1) which means that differences in the properties of LDSCs and ADSCs could not be affected by the differences in patients’ age.

Mesenchymal stem cells isolated from adipose tissue show a spindle-cell-like bipolar morphology which is typical for mesenchymal stem cells [23,26]. It has been shown that subcutaneous ADSCs are more homogenous in morphology with less variations in cell diameter up to passage 12, compared to other adipose tissue depots [23]. In our study, the morphology of LDSCs and ADSCs was very similar and typical mesenchymal-like, without significant differences observed between LDSCs and ADSCs after isolation as well as after cell passage (Figure 1). Although, some authors reported that ADSCs have consistent morphology while LDSCs did not [17], several studies showed that morphology of LDSCs is very much alike ADSCs and there were no morphological differences between those two cell types after long-term culture [13,14]. 

To be considered as stem cells, cells should fulfil some phenotypic criteria and to express so called stemness-related markers which include panel of both intracellular and surface molecules. Oct4, the most commonly examined intracellular marker, is a transcriptional factor encoded by the *POU5F1* gene. It is expressed in embryonic stem cells [27] and also in adult human stem cells [28], and is essential for pluripotency, self-renewal, proliferation and survival of mesenchymal stem cells [29]. Expression level of Oct4 in cells is reported to define the balance between differentiation and de-differentiation in stem cells [30]. In our study, both LDSCs and ADSCs expressed *POU5F1* with no statistically significant difference in expression level when compared (Figure 2f). There is a lack of data in the literature on Oct4 expression in LDSCs, however, there are some reports on expression of *POU5F1* in lipoma tissue, and it has been shown that this gene is up-regulated in lipoma compared to the normal adipose tissue [31]. 

CD44 is a transmembrane glycoprotein which is very important for cell differentiation and functions as a receptor for hyaluronic acid that is involved in cell–cell and cell-matrix interactions, mediates cell adhesion and migration, and interacts with other ligands, such as osteopontin, collagen, and matrix metalloproteinases (MMPs). Numerous studies have shown that CD44, as well as *CD44* gene, is expressed in ADSCs and is considered as one of the highly expressed positive stem cell markers in ADSCs [4,23,32,33]. In a very few studies dealing with LDSCs, it was shown that CD44 is expressed in LDSCs and there is no difference in expression level between LDSCs and ADSCs [17]. Zavan et al. [31] showed that *CD44* is up-regulated in lipoma tissue compared to normal adipose tissue. In our study, both ADSCs and LDCSs express the *CD44* (Figure 2e) although LDSCs slightly less but not significantly. The lower *CD44* expression in LDSCs may be the consequence of hypermethylation of the *CD44* gene which is the case in tumor development [34]. This finding could be taken into consideration to explain weaker differentiation potential of LDSCs as shown later. 

Endoglin (ENG) or CD105 is a type I membrane glycoprotein and is a part of the TGF beta receptor complex. It is highly expressed in ADSCs and MSCs in general, and represents one of the commonly used positive markers for their characterization [4,23,32,33,35]. In our study both LDSCs and ADSCs expressed high levels of CD105 as evaluated by flow cytometry (Figure 2a,c). There are opposite findings in literature about expression of CD105 in LDSCs. Some authors reported that expression of CD105 is similar in LDSCs and ADSCs as analyzed by flow cytometry [17], which is in accordance with our results, while others reported low CD105 expression in LDSCs [15]. This opposite finding of Tremp et al. [15] is probably due to the different type of lipoma used, lack of comparative measurement of its expression in normal ADSCs and analysis in SVF cells while our cells were characterized at passage 2. Chang et al. [36] analyzed, by flow cytometry, the expression of CD105 in cells isolated from two different types of lipoma and showed that all cells highly express CD105 (greater than 90%) with no difference between different types of lipoma samples. It has been shown that expression of CD105, and other stem cells surface markers, is low in SVF cells, but increases during cultivation and passages while isolated SVF has high expression levels of pluripotency (embryonic stem cell) markers, which decrease with cultivation and passages [37,38]. This is one of the main reasons why we used cells at passage 2 for further differentiation studies since they expressed both pluripotency markers (Oct4) as well as stem cell surface markers CD44 and CD105, at this point (Figure 2).

The ADSCs at passage 2 or 3 uniformly express stem cell markers and are morphologically a homogeneous population [26,39]. This finding is confirmed in our study as well, showing that both LDSCs and ADSCs express stem cell markers and are morphologically homogeneous at passage 2. Our results of stemness-related markers’ analysis suggest that both LDSCs and ADSCs in our study were mesenchymal stem cells, which means that both had similar phenotype and stemness state before differentiation. Senescence (or aging) of cells represents an irreversible process during which stem cells lose their stemness-related phenotype and differentiation potential, and occurs in long-term cell culture and at late cell passages [40,41,42]. ADSCs are shown to enter into senescence after passage 10 [41] and at lower extent than stem cells of other origin [42]. We used cells at early passage (P2) for differentiation studies thus avoiding the influence of cell senescence on differentiation potential.

Our results are among the first presenting detailed analysis of molecular signature of LDSCs in comparison with ADSCs, which were examined at the same time and under the same conditions. To the best of our knowledge, there are no data on expression of adipogenic- and osteogenic-related markers in stem cells isolated from lipoma tissue. We analyzed relative expression levels of genes *ADIPOQ*, *LEP*, *PPARG*, *DLK1*, *RUNX2* and *BGLAP* in LDSCs and ADSCs at passage 2, before differentiation started (Figure 3). These results are of great importance for analysis of differentiation capacity of those cells and differences between them, since these results gave us information about the basal levels of mRNA from genes that we examined during adipogenic and osteogenic differentiation. We showed that there are slight differences in all analyzed genes, however, statistically significant difference between LDSCs and ADSCs was observed for *RUNX2* and *BGLAP* only. This could explain the differences in the osteogenic differentiation dynamics between LDSCs and ADSCs noticed in our study. There is one report on adiponectin and PPAR-γ expression in sorted LDSCs (distinguished CD34+ cells) evaluated by immunocytochemical staining, and it has been shown that adiponectin expression was lower in CD34+ cultured cells from lipoma compared to CD34+ isolated from normal adipose tissue with no difference in the expression of PPAR-γ [31]. This finding correlates with our results of gene expression analysis (Figure 3c,e). Although there are no studies on gene expression in isolated cells some research groups investigated the differences in expression of *ADIPOQ*, *LEP* and *PPARG, RUNX2* and *BGLAP* in lipoma tissue and compared it with normal adipose tissue [14,31]. It has been shown that, in tissue samples, expression of *LEP* was higher while expression of *ADIPOQ* was lower in lipoma compared to normal adipose tissue, which is the expression pattern characteristic for obesity [14]. The same pattern of *LEP* and *ADIPOQ* gene expression was observed in our study in isolated cells (Figure 3d,e). Suga et al. [14], in the same study, showed that expression of *PPARG* in lipoma was not distinctly different from that in normal adipose tissue. Our results showed that *PPARG* is more expressed in ADSCs compared to LDSCs, but not statistically significant, probably due to heterogeneity among samples (Figure 3c). In the study by Zavan et al. [31], authors showed that expression of *LEP*, *ADIPOQ* and *PPARG* genes is up-regulated while *RUNX2* was less expressed in lipoma compared to normal adipose tissue, which is, except *LEP*, different from the findings in previous study, and our results on isolated cells. 

Stem cells isolated from adipose tissue have great ability to differentiate into adipocytes and therefore are promising tool in soft tissue engineering and regenerative medicine [43]. We induced both LDSCs and ADSCs into adipocytes by cultivation in adipogenic medium for 21 day. Microscopic analysis of cells (Figure 4), Oil red O staining of lipid droplets (Figure 5), adiponectin immunoexpression (Figure 6) and leptin concentration measurement in media after 21 day (Figure 7) showed that adipogenesis in LDSCs was restricted and that LDSCs have weaker capacity to differentiate into adipocytes compared to ADSCs. These findings on cellular and protein expression levels were confirmed at gene expression level as well. Adiponectin and leptin are adipokines secreted by mature adipocytes and are commonly used as markers of adipocytes [44]. In our study, *ADIPOQ*, *LEP* and *PPARG* were significantly less expressed in LDSCs compared to ADSCs in AM medium (Figure 8) with no significant difference in *LEP* and *PPARG* expression between LDSCs cultured in different media like for ADSCs. Pref-1 is a preadipocyte marker encoded by *DLK1* gene, whose expression decreases during adipogenesis with its high expression in preadipocytes and very low or absent expression in mature adipocytes [45,46,47,48]. For mature adipocytes’ formation, decreased Pref-1 level is necessary. It has been shown that Pref-1 expression is high in ADSCs before differentiation while 14 days of adipogenic differentiation it is almost non-detectable [48]. Our results showed that *DLK1* is significantly more expressed in LDSCs cultured in AM medium for 21 day compared to ADSCs in the same medium (*p* < 0.05) (Figure 8d). Regarding ADSCs, *DLK1* expression was decreased in AM medium compared to DM medium which is in accordance with the dynamics of Pref-1 expression during adipogenesis. High levels of *DLK1* could be related with lower degree of LDSCs differentiation into adipocytes in our study. There are only few publications reported adipogenic differentiation of LDSCs. Suga et al. [14] reported that there were no differences in the capacity for adipogenic differentiation of LDSCs compared to ADSCs, at passage 1, as evaluated by lipid droplets staining and quantification. By the same analyses employed, other studies revealed that high lipid content was observed in differentiated LDSCs [15] and that cells were differentiated into adipocytes successfully, similar to ADSCs [16], but no comparative results with normal ADSCs were shown in those two studies. The ability of LDSCs to differentiate into adipocytes at early as well as late passages was reported to be similar as of ADSCs, evaluated by lipid droplets presence and staining [13]. Our results are more complex since we assessed the adipogenic differentiation potential of both LDSCs and ADSCs at different levels and by different methods. This study is, according to our knowledge, the first dealing with the adipogenic differentiation of LDSCs by analyzing the markers of this process on several levels—cellular, molecular and gene, but in the same time comparing that with ADSCs isolated from normal tissue. According to the adipogenesis-related gene expression signature as well as phenotypic changes and protein levels in LDSCs and ADSCs after 21 day of adipogenic differentiation, compared to the cells at day 0 (passage 2), we can conclude that adipogenesis was not complete in LDSCs and differentiation capacity of LDSCs compared to ADSCs was significantly lower.

Adipose-derived stem cells have a great potential to differentiate into osteoblasts and therefore represent a promising tool in bone tissue engineering for making bone grafts with different biomaterials to regenerate and repair the bone defects [23,49,50]. It has been shown that stem cells from subcutaneous adipose tissue show good osteogenic differentiation potential at passage 2 [23], that we also used in our study. After 16 days of osteogenic differentiation, phenotypic changes in both LDSCs and ADSCs cultured in OS medium were observed so that cells became more epithelial-like and less fibroblastic-like, and accumulation of inorganic matrix components was noticed (Figure 9), slightly more in ADSCs culture. No significant difference was found in ARS staining between LDSCs and ADSCs but slightly darker Von Kossa staining was observed in ADSCs. 

Osteoprotegerin (OPG) is secreted by osteoblasts, is a marker of their functional state since it is important for regulation of osteoblast-osteoclast homeostasis [51], promotes pre-osteoblasts and matrix maturation [52] and its secretion is up-regulated during osteogenic differentiation in vitro [53]. In our study, significant difference in osteoprotegerin (OPG) secretion, measured by ELISA, between LDSCs and ADSCs cultured in OS medium for 16 days, was noticed with higher OPG concentration found in ADSCs compared to LDSCs (Figure 11). 

Differences between LDSCs and ADSCs in osteogenic differentiation capacity were also observed at gene expression level in our study. *RUNX2* expression increased in both cell cultures during differentiation so it was higher in ADSCs than in LDSCs at day 8 and higher in LDSCs than ADSCs at day 16 (Figure 12a,b; Table 2). RUNX2 is a transcription factor involved in osteogenesis and is essential for osteoblast differentiation [54,55,56]. RUNX2 promotes osteoblast differentiation at an early stage, by inducing the expression of genes for bone matrix proteins such as osteopontin, osteocalcin, collagen type 1, etc., but also its high expression can inhibit osteoblast differentiation at a late stage [54,57]. In order to achieve osteoblast maturation, it is necessary that RUNX2 is initially upregulated to induce the expression of bone matrix proteins, but its expression should then decrease to enable the process of differentiation into mature osteoblasts to be continued. However, if RUNX2 is constantly upregulated, then osteoblasts maturation cannot be achieved. In our study, *RUNX2* is upregulated in LDSCs after 16 days, compared to ADSCs, which could be one of the reasons for lower expression of *BGLAP* in LDSCs compared to ADSCs. It was reported that OPG was overexpressed in pre-osteoblasts when RUNX2 was downregulated while osteoblast differentiation was arrested in RUNX2 overexpressing cells [52]. Our results are similar with these findings, since LDSCs have higher expression of *RUNX2* but lower expression of OPG, compared to ADSCs at day 16 of osteogenic differentiation where *RUNX2* expression was down-regulated and OPG was up-regulated. Osteocalcin is a protein secreted by mature osteoblasts and plays important role in bone calcification and mineralization of MSCs during osteogenic differentiation [58,59]. It was reported that *BGLAP* expression begins to increase after 14 days of osteogenic differentiation of MSCs [58,59]. In our study, *BGLAP* expression increased during differentiation in both cultures and it was higher in differentiated ADSCs than LDSCs after 16 days (Figure 12c,d; Table 2). Both LDSCs and ADSCs in our study had characteristic osteoblast-like phenotype after 16 days of osteogenic induction but, based on osteogenic genes’ expression dynamics, osteogenesis was at different stages in those cells and one of the reasons could be higher *RUNX2* expression in LDSCs at passage 2 (Figure 3a). 

There are very few publications with data about osteogenic differentiation of stem cells isolated from lipoma tissue. Makiguchi et al. [16] showed that ADSCs isolated from lipoma tissue can differentiate into osteoblasts after 21 days in OS media, as evaluated by alkaline phosphatase activity and calcium concentration measurement. It was also reported by Lin et al. [13] that LDSCs can be successfully differentiated into osteoblasts, but only Von Kossa staining of calcium deposits was performed in this study. No other markers or details on osteogenic differentiation of these cells were reported. However, there are numerous reports on lipoma tissue ossification observed in different parts of the body [8,16,18,19,60,61,62,63] and this phenomenon could be explained by the existence of stem cells in lipoma tissue that may differentiate into osteoblasts and chondrocytes. 

Upregulated expression of *RUNX2* in LDSCs, in our study, could be also considered as a reason why adipogenic differentiation of LDSCs was at lower extent since it has been shown by other authors that overexpression of RUNX2 inhibits adipogenesis [55,64,65]. Taking this finding together with the previously considered higher *RUNX2* expression before differentiation, at day 0 (passage 2) in LDSCs compared to ADSCs, it is a significant indication that *RUNX2* expression, together with *DLK1* expression pattern, is an important mechanism for suppressing LDSCs differentiation capabilities. On the other side, higher expression of *RUNX2* in LDSCs may suggest potential osteogenic capacity of those cells, which could be one of the possible mechanisms of bone and cartilaginous structures’ formation in the cases of aforementioned osteolipoma, since higher expression of RUNX2 is necessary at an early stage of both osteogenesis and chondrogenesis.

## 5. Conclusions

Our study is among the first that provides detailed analysis of stem cells isolated from lipoma and comparison with the stem cells isolated from normal adipose tissue, on cellular and molecular levels. Results of our study suggest that both LDSCs and ADSCs were mesenchymal stem cells with similar phenotype and stemness state for potential differentiation. According to analyzes of adipogenesis-related markers at cellular and molecular level and microscopic analysis after 21 day of adipogenic differentiation we can conclude that differentiation capacity of LDSCs compared to ADSCs was significantly lower. Analysis of osteogenesis-related markers revealed that both types of cells had characteristic osteoblast-like phenotype after 16 days of osteogenic induction but were at different stages of osteogenesis. Differences between LDSCs and ADSCs, after isolation from the tissue, are probably due to the distinct molecular signature and their commitment in the tissue that will governs their different capacity and fate during adipogenic and osteogenic induction in vitro. These results provide new insights into the cellular and molecular basis of lipoma etiopathogenesis and imply that the potential use of LDSCs in tissue engineering and regenerative medicine should be re-considered or at least other ways of using these cells for regeneration purposes should be discussed.

## Figures and Tables

**Table 1:** Relative expression of *ADIPOQ*, *LEP*, *PPARG* and *DLK1* in LDSCs and ADSCs at day 21 of adipogenic differentiation, normalized to *GAPDH* and compared to the cells at P2 (day 0). Results are expressed as mean values ± SD. Asterisks represent statistical significance compared to P2 (day 0).

Cell Type	*ADIPOQ*			*LEP*		
	P2 (Day 0)	21 Day		P2 (Day 0)	21 Day	
		AM	DM		AM	DM
**LDSCs**	0.033 ± 0.033	12.530 ± 7.31 *	0.400 ± 0.35	0.0130 ± 0.011	0.03 ± 0.012 *	0.030 ± 0.025
**ADSCs**	0.061 ± 0.019	993.84 ± 174.09 ***	0.515 ± 0.45	0.0041 ± 0.003	0.46 ± 0.41 ***	0.025 ± 0.006 **
	***PPARG***			***DLK1***		
	**P2 (Day 0)**	**21 Day**		**P2 (Day 0)**	**21 Day**	
		**AM**	**DM**		**AM**	**DM**
**LDSCs**	0.159 ± 0.151	0.30 ± 0.187	0.32 ± 0.247	0.32 ± 0.725	6.86 ± 4.38 *	1.50 ± 1.73
**ADSCs**	0.250 ± 0.188	4.92 ± 3.50 *	0.42 ± 0.315	0.10 ± 0.105	0.57 ± 1.13	2.61 ± 4.92

* *p* < 0.05, ** *p* < 0.01, *** *p* < 0.001.

**Table 2:** Relative expression of *RUNX2* and *BGLAP* in LDSCs and ADSCs at days 8 and 16 of osteogenic differentiation, normalized to *GAPDH* and compared to the cells at P2 (day 0). Results are expressed as mean values ± SD. Asterisks represent statistical significance compared to P2 (day 0).

Cell Type	*RUNX2*				
	P2 (Day 0)	8 Days		16 Days	
		OS	DM	OS	DM
**LDSCs**	1.620 ± 0.622	2.156 ± 1.133	2.27 ± 1.390	7.194 ± 4.76 *	6.478 ± 5.83
**ADSCs**	1.013 ± 0.214	2.670 ± 1.799	4.01 ± 1.747 *	5.685 ± 1.29 ***	8.960 ± 3.35 **
	***BGLAP***				
	**P2 (Day 0)**	**8 Days**		**16 Days**	
		**OS**	**DM**	**OS**	**DM**
**LDSCs**	0.23 ± 0.039	0.312 ± 0.091 *	0.368 ± 0.099 *	0.323 ± 0.072 *	0.388 ± 0.081 **
**ADSCs**	0.33 ± 0.085	0.395 ± 0.091	0.503 ± 0.147	0.554 ± 0.155 *	0.600 ± 0.062 **

* *p* < 0.05, ** *p* < 0.01, *** *p* < 0.001.
